# Engineering a lipase B from *Candida antactica* with efficient perhydrolysis performance by eliminating its hydrolase activity

**DOI:** 10.1038/srep44599

**Published:** 2017-03-20

**Authors:** Xu-Ping Wang, Peng-Fei Zhou, Zhi-Gang Li, Bo Yang, Frank Hollmann, Yong-Hua Wang

**Affiliations:** 1 School of Food Sciences and Engineering, South China University of Technology, Wushan road 381, Tianhe District, 510640 Guangzhou, P.R. China; 2 School of Bioscience and Bioengineering, South China University of Technology, Daxuecheng, Panyu District, 510006 Guangzhou, P.R. China; 3 Department of Biotechnology, Delft University of Technology, Van der Maasweg 9, 2629HZ Delft, The Netherlands

## Abstract

A Ser105Ala mutant of the lipase B from *Candida antarctica* enables ‘perhydrolase-only’ reactions. At the example of the chemoenzymatic Baeyer-Villiger oxidation of cyclohexanone, we demonstrate that with this mutant selective oxidation can be achieved in deep eutectic solvent while essentially eliminating the undesired hydrolysis reaction of the product.

In 1899, Adolf von Baeyer and Victor Villiger first reported the transformation of ketones into esters or lactones using peracids^1^. Ever since then the Baeyer-Villiger (BV) oxidation represents one of the most-studied reactions in organic synthesis^2^,^3^. Particularly, the formation of ε-caprolactone is of interest due to its importance as polymer building block^4^. Commonly, peracids such as *m*-chloroberbenzoic acid or peracetic acid are used as stoichiometric reagents in BV-oxidation^5^,^6^,^7^.

Due to the poor atom efficiency of this methodology catalytic methods are now in focus of current research. Highly stereoselective BV-oxidations using so-called Baeyer-Villiger Monooxygenases (BVMO) are known^8^,^9^,^10^,^11^,^12^,^13^. However, due to their cofactor-dependency and sometimes low intrinsic stability, BVMOs are not yet practical catalysts^14^. In addition, BVMOs also can suffer from product inhibition impairing their catalytic efficiency. Bornscheuer and coworkers solved this by the lipase-catalyzed oligomerization of the corresponding lactones which may not always be the desired solution (product)^3^. Alternatively, chemoenzymatic BV-oxidations using hydrolases are gaining relevance in organic synthesis^15^. These systems utilize hydrolase-catalyzed formation of per acids (in a promiscuous ‘perhydrolase’ activity) followed by spontaneous BV-oxidation (Fig. 1).

One major drawback of this approach however lies with the intrinsic activity of hydrolases with the products of interest. Esters are preferentially hydrolyzed by hydrolases leading to the undesired formation of ω-hydroxy acids. Hence, use of simple (aqueous) H_2_O_2_ is not possible but anhydrous reagents such as urea-H_2_O_2_ adducts have to be used^16^.

Recently, we have reported a mutant of the lipase from *Penicillium camembertii* where in the catalytic triad was disrupted by mutating Ser145 to an alanine^17^. Obviously this mutant showed no more hydrolase activity but some residual perhydrolase activity. The proposed mechanism for the Ser145Ala mutant involves activation of H_2_O_2_ by the remaining histidine^17^. In continuation of this work we suggested that a similar mutant of the well-known lipase B from *Candida Antarctica* (CalB) might exhibit a similar reaction pattern. This mutant has been investigated in detail by Hult and coworkers for various ‘unnatural’ reactions such as aldol^18^ and Michael-type reactions on α,β-unsaturated carbonyl groups^19^,^20^. Therefore, we synthesized the analogous CalB mutant missing the catalytic serine residue of the catalytic triad (Ser105) and replaced it by an alanine.

## Results and Discussion

The mutant enzyme was heterologously expressed and purified to homogeneity (as judged by SDS gel analysis, Fig. SI). Then, we proceeded with the evaluation of Ser105Ala for selective BV-oxidation of cyclohexanone. It is worth mentioning that control experiments using a thermally inactivated Ser105Ala (under otherwise identical conditions) revealed no significant Baeyer-Villiger oxidation activity. Also, no conversion was observed in the absence of free carboxylic acids (data not shown).

Quite expectedly, Ser105Ala exhibited almost no hydrolytic activity for example on ε-caprolactone whereas the wild type enzyme smoothly hydrolyzed this substrate (Fig. S2). Encouraged by this we proceeded to the chemoenzymatic BV-oxidation of cyclohexanone comparing both enzyme variants (Fig. 2).

Surprisingly, the ε-caprolactone formation rate was only two times higher in case of wt-CalB as compared to the Ser105Ala mutant. Furthermore, accumulation of ε-caprolactone continued for at least 48 h in case of the mutant whereas it stopped after 3 h using the wt-CalB. One plausible explanation for this may be acidification of the reaction medium due to lactone hydrolysis.

More importantly, the acid to lactone ratio was inverted from 2:1 (in case of the wt-CalB) to 1:2.5 in case of the Ser105Ala-mutant. In other words, the selectivity of the overall reaction was improved about 5-fold. The hydrolysis product observed in case of the Ser105Ala-reaction can most likely be attributed to spontaneous hydrolysis of the ε-caprolactone under the current reaction conditions. Indeed, control reactions testing the stability of ε-caprolactone in the reaction medium gave essentially a comparable hydrolysis rate (Fig. S2). Therefore, we are confident that this issue can be overcome in future research by e.g. *in situ* extraction of the lactone into a suitable organic phase thereby preventing the undesired hydrolysis.

**Figure 1:**
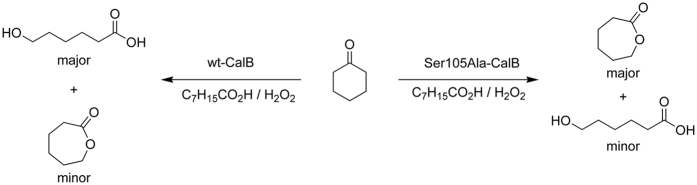
The chemoenzymatic BV-oxidation. The hydrolase mediates the perhydrolysis reaction of octanoic acid to peroctanoic acid, which then spontaneously transforms cyclohexanone to ε-caprolactone (desired reaction). Hydrolysis of the lactone product, catalyzed by the hydrolase, represents the undesired side-reaction.

**Figure 2:**
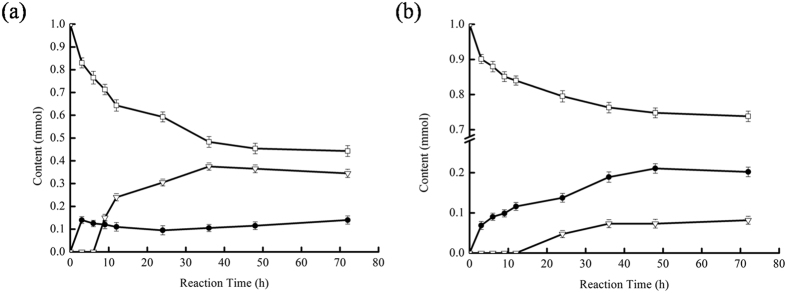
Products formation of ε-caprolactone (●) and 6-hydroxycaproic acid (△) and reducing of cyclohexanone (□) using wt-CalB (**a**) and Ser105Ala (**b**). General conditions: cyclohexanone (1 mmol), 30% aq. H_2_O_2_ (2 mmol), octanoic acid (1 mmol), 5 mg CalB (wild-type or S105A), H_2_O (1 mL), *n*-hexane (2 mL). T = 40 °C.

It should be mentioned that, both the wt-CalB and Ser105Ala exhibited comparably poor stability under the reaction conditions chosen in Fig. 1. Essentially, after 48 h no more product formation was observable, which we attribute to a loss in catalytic activity of the biocatalyst used.

Inspired by a recent contribution reporting the beneficial effect of deep eutectic solvent (DES) on the activity and stability of lipase^21^, we decided to evaluate a range of DES as alternative solvents for the chemoenzymatic BV-oxidation of cyclohexanone (Table 1).

Pleasingly, using DES generally improved the overall conversion of cyclohexanone. Particularly, ChCl/sorbitol increased the product formation by almost a factor of two, both for the wt- and the mutant enzyme. Again, control reactions with thermally inactivated enzymes yielded no detectable conversion of the starting material.

Encouraged by this, we further elucidated ChCl/sorbitol as ‘performance additive’ for a range of chemoenzymatic BV-oxidations (Table 2).

The results shown in Table 2, confirm our previous observation that Ser105Ala enables significantly more selective reactions. In essence, hydrolysis was not observed with Ser105Ala. Interestingly, however, also the DES appeared to have a beneficial effect on the chemoselectivity by suppressing the undesired hydrolysis reaction. Currently, we are lacking a plausible explanation for this phenomenon. Possibly, the DES also influenced the water activity.

In conclusion, Ser105Ala indeed is a ‘perhydrolase only’ enzyme. Expectedly, removal of the catalytic triad Ser eliminated the enzyme’s hydrolytic activity. However, the perhydrolase activity was largely maintained. Admittedly, the current mutant is not suitable for large-scale preparative applications and further improvements of the specific enzyme activity via structure-guided protein engineering are currently underway in our laboratory. Despite the preliminary character of this study the product to enzyme ratio achieved was already roughly 10:1. Therefore, we are confident that a combination of enzyme- and reaction-engineering will result in a practical protocol for the synthesis of lactones.

## Experimental Section

### Material

*Escherichia coli* DH5α and plasmid pGAPZαA (Invitrogen, USA) were used as cloning host and vector, respectively. *Pichia pastoris* X-33 (Life technology, China) strain was used for expression. Polymerase and DNA restriction endonuclease were purchased from Takara biotechnology Co. Ltd (Dalian, China). Chemicals used in this study were purchased from Aladdin^®^ Chemistry Co. Ltd (Shanghai, China) at the highest purity available.

### CalB cloning and mutagenesis

The codon optimized the lipase B from *Candida Antarctica* (CalB) gene was synthesized by Shenggong biotechnology company (Shanghai, China). The resulted CalB gene was inserted into the pGAPZαA plasmid to get the expression vector pGAPZαA-CalB. Site-directed mutagenesis was carried out by site-directed mutagenesis following the QuikChange protocol (Stratagene, USA). Primers for mutant construction were designed by QuikChange Primer Design. The Ser105Ala mutant was confirmed by DNA sequencing in BGI (Shenzhen, China).

### Protein Expression and Purification

Expression of CalB and mutant was performed as described previously^17^. They purified using Ion Exchange Chromatography (DEAE-Sephadex, GE Healthcare, China) and freeze dried. The purified proteins were analyzed by SDS-PAGE. Protein concentrations were determined by the Bio-Rad Protein Assay (Bio-Rad Laboratories, Inc, USA).

### Preparation of DESs

The deep eutectic solvents (DESs) were prepared according to the method^22^. The corresponding solid components of the desired DESs in the correct proportion were placed in a 250 mL round-bottom flask. The mixtures were heated at 100 °C under rotary evaporation until a homogeneous transparent liquid was formed.

### Baeyer-Villiger oxidation reaction in water/*n*-hexane

Reactions mixture contained cyclohexanone (1 mmol), octanoic acid (1 mmol), *n*-hexane (2 mL), phosphate buffer (20 mmol pH 6.0, 1 mL) and lipase (wt-CalB, Ser105Ala or thermally inactivated CalB, 5 mg), 30% aq. H_2_O_2_ was added in 10 portions at 5 h intervals (total 2 mmol). The reaction was carried out at 40 °C for 72 h with magnetic stirring at 500 rpm. Extraction of the sample was done with ethyl acetate and removed water by anhydrous Na_2_SO_4_.

### Baeyer-Villiger oxidation reaction in DESs

Reactions mixture contained ketone (1 mmol), octanoic acid (1 mmol), DES (1.2 g), H_2_O (0.3 mL) and lipase (wt-CalB or Ser105Ala, 5 mg), 30% aq. H_2_O_2_ was added in 10 portions at 5 h intervals (total 2 mmol). The reaction was carried out at 40 °C for 48 h. Other reaction conditions and the sample treated method as above.

### Hydrolysis of ε-caprolactone in reaction medium

To verify whether Ser105Ala could hydrolyze ε-caprolactone in the reaction medium, ε-caprolactone (1 mmol), octanoic acid (1 mmol), *n*-hexane (2 mL), phosphate buffer (20 mmol pH 6.0, 1 mL) and lipase (wt-CalB, Ser105Ala or thermally inactivated CalB, 5 mg) were mixed in 10 mL conical flask. Then 30% aq. H_2_O_2_ was added in 10 portions at 5 h intervals (total 2 mmol). The mixture was carried out at 40 °C for 24 h with magnetic stirring at 500 rpm. The thermally inactivated CalB was prepared by boiling CalB in water for 2 h. Extraction of the sample was done with ethyl acetate and removed water by anhydrous Na_2_SO_4_.

### Compounds Analysis

Gas chromatographic analyses were carried out with an Agilent Technology model 7890 GC-instrument equipped with a WAX 30 m × 0.25 mm × 2.0 μm column. A temperature program was used to keep the samples in a column oven at 60 °C for 1 min, then increased to 113 °C at 5 °C/min, increased to 190 °C at 20 °C/min, increased to 240 °C at 10 °C/min for 5 min. The split ratio was 30:1. The injector and the flame ionization detector temperatures were set at 250 and 280 °C, respectively. Peaks in GC chromatograms were identified by comparison of their retention times with reference standards.

### Calculations


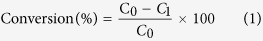



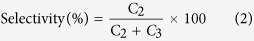


where C_0_ (mM) refers to the concentration of ketones (cyclobutanone, cyclopentanone, cyclohexanone and 4-heptanone) at t = 0 h; C_1_ (mM) refers to ketones at t = 48 h. C_2_ (mM) refers to the concentration of lactones and ester (γ-butyrolactone, δ-valerolactone, ε-caprolactone and propyl butyrate); C_3_ (mM) refers to the concentration of acids (6-hydroxycaproic acid, 5-hydroxyvaleric acid, 4-hydroxy-butanoic acid and butyrate).

## Additional Information

**How to cite this article:** Wang, X.-P. *et al*. Engineering a lipase B from *Candida antactica* with efficient perhydrolysis performance by eliminating its hydrolase activity. *Sci. Rep.* **7**, 44599; doi: 10.1038/srep44599 (2017).

**Publisher's note:** Springer Nature remains neutral with regard to jurisdictional claims in published maps and institutional affiliations.

## Supplementary Material

Supplementary Information

## Figures and Tables

**Table 1:** BV-oxidation of cyclohexanone by wt-CalB and Ser105Ala in various DESs.

Entry	Solvent	Conversion (%)	
		wt-CalB	Ser105Ala
1	Water: *n*-hexane (1:2)	55	24
2	ChCl: urea (1:2)	76	26
3	ChCl: ethanediol (1:2)	81	29
4	ChCl: glycerol (1:2)	85	33
5	ChCl: xylitol (1:1)	89	40
6	ChCl: sorbitol (1:1)	92	47

General conditions: cyclohexanone (1 mmol), 30% aq. H_2_O_2_ (2 mmol), octanoic acid (1 mmol), 5 mg CalB (wild-type or S105A), DES (1.2 g), H_2_O (0.3 mL). T = 40 °C, time 48 h.

**Table 2:** Substrate Scope of wt-CalB and Ser105Ala catalyzed BV-oxidation.

Entry	Substrate	Product	wt-CalB		Ser105Ala	
			Conversion (%)	Selectivity (%)	Conversion (%)	Selectivity (%)
1	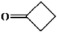	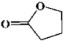	99	93	99	100
2	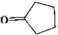	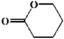	95	48	51	97
3	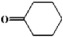	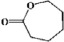	92	46	47	99
4	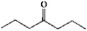	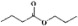	79	35	38	96

General conditions: ketone (1 mmol), 30% aq. H_2_O_2_ (2 mmol), octanoic acid (1 mmol), 5 mg CalB (wild-type or S105A), ChCl/sorbitol (1.2 g), H_2_O (0.3 mL). T = 40 °C, time 48 h.
