# Supplementary material for: Engineering a lipase B from Candida antactica with efficient perhydrolysis performance by eliminating its hydrolase activity
Source: Sci Rep. 2017 Mar 20;7:44599. doi: 10.1038/srep44599 (PMC5357956; doi:10.1038/srep44599)
Supplement: Supplementary Information [file srep44599-s1.pdf]

Supporting Information for:

Engineering a lipase B from *Candida antactica* with efficient perhydrolysis performance by eliminating its hydrolase activity

Xu-Ping Wang[1], Peng-Fei Zhou[2], Zhi-Gang Li[2], Bo Yang[2], Frank Hollmann[3] and Yong-Hua Wang[1,]*

[1] School of Food Sciences and Engineering, South China University of Technology, Wushan road 381, Tianhe District, 510640 Guangzhou, P.R. China
[2] School of Bioscience and Bioengineering, South China University of Technology, Daxuecheng, Panyu District, 510006 Guangzhou 510006, P.R. China
[3] Department of Biotechnology, Delft University of Technology, Van der Maasweg 9, 2629HZ Delft, The Netherlands

*Corresponding author:
yonghw@scut.edu.cn (Yong-Hua Wang)

Other authors:
wangxuping88@163.com (Xu-Ping Wang)
bipfzhougz@mail.scut.edu.cn (Peng-Fei Zhou)
bizgl@scut.edu.cn (Zhi-Gang Li)
yangbo@scut.edu.cn (Bo Yang)
F.Hollmann@tudelft.nl (Frank Hollmann)

Table of Contents

Figure S1. SDS/PAGE of purified wt-CalB and Ser105Ala mutant
Figure S2. Hydrolysis of ε-caprolactone in reaction medium

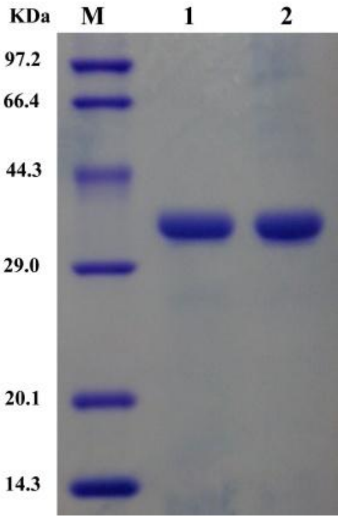

Figure S1. **SDS/PAGE of purified wt-CalB and Ser105Ala mutant.** Lane 1, wt-CalB; Lane 2, Ser105Ala mutant.

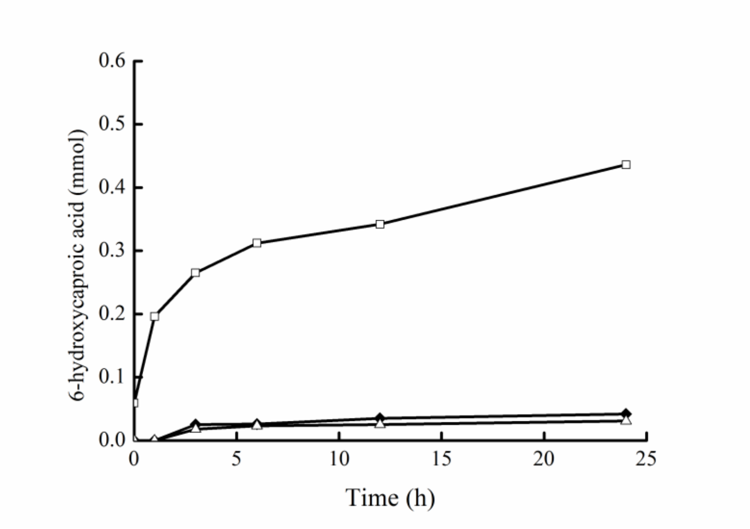

Figure S2. **Hydrolysis of ε-caprolactone in reaction medium.** (□) wt-CalB, (◆) Ser105Ala mutant, (△) Thermally inactivated CalB. General conditions: ε-caprolactone (1 mmol), 30% aq. H_2O_2 (2 mmol), octanoic acid (1 mmol), 5 mg CalB (wild-type or S105A), H_2O (1 mL), *n*-hexane (2 mL). T = 40 °C.
